# Interdependencies between acoustic and high-speed videoendoscopy parameters

**DOI:** 10.1371/journal.pone.0246136

**Published:** 2021-02-02

**Authors:** Patrick Schlegel, Andreas M. Kist, Melda Kunduk, Stephan Dürr, Michael Döllinger, Anne Schützenberger

**Affiliations:** 1 Department of Head & Neck Surgery, David Geffen School of Medicine, University of California Los Angeles (UCLA), Los Angeles, California, United States of America; 2 Dep. of Otorhinolaryngology, Div. of Phoniatrics and Pediatric Audiology, University Hospital Erlangen, Friedrich-Alexander-University Erlangen-Nürnberg, Erlangen, Germany; 3 Dep. of Communication Sciences and Disorders, Louisiana State University, Baton Rouge, Louisiana, United States of America; University of Colorado School of Medicine, UNITED STATES

## Abstract

In voice research, uncovering relations between the oscillating vocal folds, being the sound source of phonation, and the resulting perceived acoustic signal are of great interest. This is especially the case in the context of voice disorders, such as functional dysphonia (FD). We investigated 250 high-speed videoendoscopy (HSV) recordings with simultaneously recorded acoustic signals (124 healthy females, 60 FD females, 44 healthy males, 22 FD males). 35 glottal area waveform (GAW) parameters and 14 acoustic parameters were calculated for each recording. Linear and non-linear relations between GAW and acoustic parameters were investigated using Pearson correlation coefficients (PCC) and distance correlation coefficients (DCC). Further, norm values for parameters obtained from 250 ms long sustained phonation data (vowel /i/) were provided. 26 PCCs in females (5.3%) and 8 in males (1.6%) were found to be statistically significant (|corr.| ≥ 0.3). Only minor differences were found between PCCs and DCCs, indicating presence of weak non-linear dependencies between parameters. Fundamental frequency was involved in the majority of all relevant PCCs between GAW and acoustic parameters (19 in females and 7 in males). The most distinct difference between correlations in females and males was found for the parameter *Period Variability Index*. The study shows only weak relations between investigated acoustic and GAW-parameters. This indicates that the reduction of the complex 3D glottal dynamics to the 1D-GAW may erase laryngeal dynamic characteristics that are reflected within the acoustic signal. Hence, other GAW parameters, 2D-, 3D-laryngeal dynamics and vocal tract parameters should be further investigated towards potential correlations to the acoustic signal.

## Introduction

Phonation begins with an airstream, rising from the lungs, setting the vocal folds located in the larynx in motion. The vocal folds subdivide this airstream in a series of flow pulses which are further modulated in the vocal tract until exiting through the mouth and being perceived as acoustic signal [1, 2]. It is logical to assume that relations between vocal fold oscillation characteristics and acoustic sound quality should exist. Uncovering such relations would highly improve treatment possibilities of voice disorders, since this knowledge will guide physicians in deciding what specific oscillation characteristic needs to be addressed in order to improve certain acoustic quality features.

Due to different underlying disorders the process of voice production can be impaired in a variety of ways. In this work, we divide voice disorders in two groups: organic dysphonias (OD) and functional dysphonias (FD) [3]. Whilst signs of ODs are always (visible) laryngeal anatomical changes, FD is a diagnosis of exclusion due to no underlying anatomical/tissue related (visible) changes are ascertainable [4]. A voice disorder classified as FD may also have purely psychological etiology [5]. It is important to note that some uncertainty surrounds the term FD. First, the exact boundary between ODs and FDs is not always absolute, since organic pathologies may eventually result in functional disorders [3], being named a secondary functional dysphonia. Second, subcategories of FD are not entirely standardized and often reflect clinician’s supposition and bias in practice [6]. However, in this study the subjects with FD diagnosis had no organic pathologies at the time of recording, i.e. only the so called primary functional dysphonia was considered.

In patients with voice disorders, the acoustic signal is altered. In many cases, this is due to impairments in the vocal fold oscillations [7, 8]. It is assumed that there are three main vocal fold dynamical characteristics that foster healthy voice quality [9–11]: vocal fold oscillations are assumed to be (A) symmetric, (B) periodic and (C) exhibit a closed state during oscillations.

For instance, vocal fold asymmetry [12, 13] and aperiodicity [14] have been linked to perceived audible roughness; incomplete glottis closure is associated with vocal fatigue and a breathy voice [7, 8]. Better understanding the relations between features of vocal fold oscillations and their effects on the acoustic signal could be of great benefit in clinic settings: If auditory-perceptual symptoms can be traced back to specific vocal folds disorders or specific patterns of vocal fold oscillations, this may lead to improvement in patient’s voice by directly treating underlying cause. Hence, finding relations between acoustic signal quality and vocal fold oscillation characteristics would provide further insight into fundamental connections in voice production and would eventually allow treatments tailored to the individual patient’s needs.

**Fig 1:**
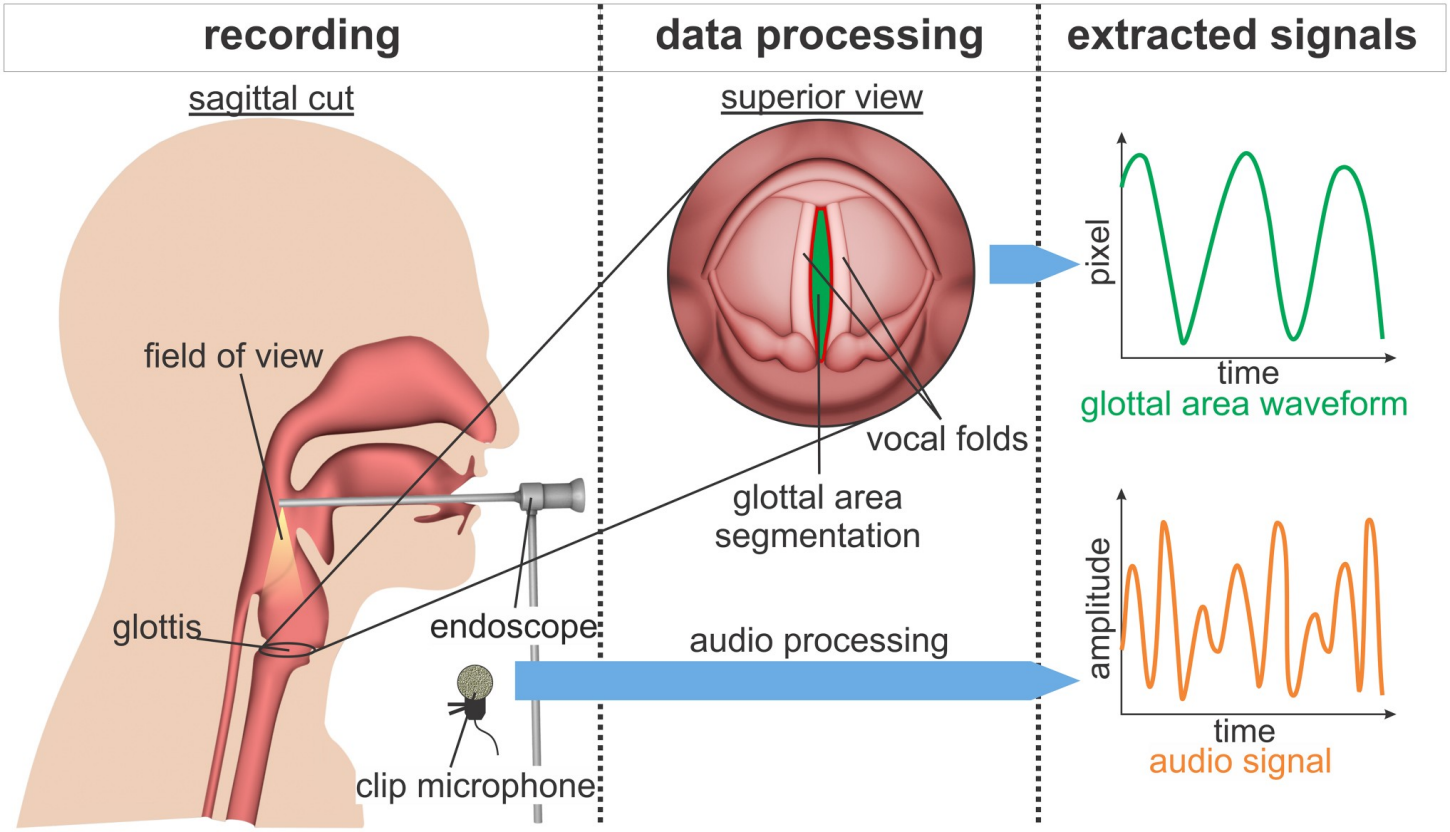
Parallel recording of acoustic and HSV data with subsequent extraction of signals.

One powerful tool for investigating vocal fold oscillations is high-speed videoendoscopy (HSV) [15–17]. As illustrated in Fig 1, during rigid-endoscope HSV data collection, as performed in this study, an endoscope is inserted in the mouth of the subject, to record the vocal fold oscillations. The oscillation frequency of the vocal folds lies between 80 and 400 Hz during normal phonation [3]. With HSV recording frame rates between 4,000 fps and 20.000 fps these oscillation frequencies are easily captured [7, 18], leading to a thorough recording of oscillation characteristics during each glottis cycle.

From the resulting HSV data, different types of signals can be extracted, such as vocal fold trajectories [19], Phonovibrograms [20] and the "Glottal Area Waveform" (GAW) [21]. The GAW describes the changing area between the vocal folds, i.e. the glottal area, over time. The GAW reaches maxima during maximum opening of the glottis and minima during the closed phase. Also, synchronous recording of the acoustic signal is possible and often put into practice [22–24] as it was done in this work.

Based on the extracted acoustic and GAW signals various parameters can be calculated, describing different features of the signals reflecting different features of the voice production process. A great number of parameters have been introduced [9, 25], but norm-values for many parameters are still missing due to a variety of reasons [26–29]. Widely used parameters such as Jitter and Shimmer describe period irregularity in fundamental frequency and amplitude in the signal. Increased values of these parameters are e.g. associated with hoarseness if they were calculated on acoustic signals [30]. However, given norm values for Jitter (in this case Jitter Percent) differ, with one study stating "healthy" values of around 0.25% for females and males while producing the vowel /a/ [31] whereas another study considers values as high as 0.53% for younger and 0.84% for older males phonating the vowel /a/ as healthy [32]. Such differences may be related to inadequate subject recruitment in these studies or other variabilities in the data collection process. Also in studies employing HSV data different factors appeared to be influencing these parameters such as recording frame rate [27], camera resolution [28] or sequence length [29]. Hence, norm value tables for HSV parameters to aid in objective separation of healthy and disordered voices are needed.

To this date, various works have investigated relations between vocal fold movements and resulting acoustics. However, often only linear relations were explored [33–36] or data from a small number of subjects (N ≤ 20) was used [33, 35–37]. Some relations between vocal fold oscillations and resulting acoustic signal are known with the most obvious one being the strong correlation between fundamental frequency of the vocal fold oscillations and the fundamental frequency of the resulting acoustic signal in sustained phonation. Other examples include connections between insufficient closure of the vocal folds during phonation and perceived hoarseness in the acoustic signal or the “force” with which the vocal folds collide and the acoustic amplitude [7, 8]. The fundamental frequency (F_0_) at which the subject phonates is another factor that may influence acoustic and GAW parameters. For instance, period perturbation measurements in the GAW may be influenced by F_0_ due to the lower sampling rate of GAW signals and a changing F_0_ may affect more complex parameters such as noise measurements [30].

This study investigated linear and non-linear relations between GAW and acoustic data for a large number of subjects and parameters. Female and male subjects with normal voices formed the healthy voice group, and subjects who had been diagnosed with FD formed the voice disordered group. The influence of F_0_ on the other parameters considered in this work was of particular interest, since parameters that are strongly affected by F_0_ may require a correction of this influence. Further, we used our collected data to provide preliminary norm values of parameters obtained from 250 ms long sustained phonation data (vowel /i/). The aims of this work are:

1. Create a set of norm-values for all investigated parameters that differentiate females and males with normal voices from subjects with diagnosis of FD for the given recording settings.
2. Find parameters that are influenced by F_0_
3. Determine the linear and non-linear relations between GAW and acoustic parameters

## Methods

HSV recordings (N: 351) with simultaneously recorded acoustic signal (time-synchronized) were used for data evaluation. This data (without the acoustic recordings) was already used in a previous study applying machine learning approaches for classification purposes [38]. All 351 acoustic recordings were unanimously rated by three experts on ordinal scales (0 to 2) for signal noise and background noise: 0 was chosen as the best rating (no signal noise / background noise) and 2 as the worst (strong signal noise / background noise). Only recordings that had signal noise and background noise rated at 1 or 0, were used in further analysis, leading to final set of 250 combined HSV-acoustic recordings from female and male subjects for further analyses.

The 250 combined HSV-acoustic recordings were divided into four groups depending on their gender and health status, Table 1. All recordings were taken under clinical conditions using a Photron Fastcam MC2 camera (frame rate: 4000 fps, resolution: 512×256 pixels, 70° rigid endoscope). The acoustic signal was simultaneously recorded using a clip microphone (pentax model #7175–6000, Lapel Microphone, Audio Technica ASP-0091, sampling rate: 40 kHz). All subjects phonated the vowel /i/ at their habitual pitch and loudness level (sustained phonation). From each combined HSV-acoustic recording, a section of 250 ms of sustained phonation was selected.

**Table 1:** Number of combined HSV-acoustic datasets and subject range of age for each group (healthy females (N_F_), females with FD (FD_F_), healthy males (N_M_) and males with FD (FD_M_)).

	Healthy	Disordered
**Females**	124 (N_F_) age range 18–64 (mean: 22)	60 (FD_F_) age range 20–79 (mean: 49)
**Males**	44 (N_M_) age range 21–30 (mean: 25)	22 (FD_M_) age 26–83 range (mean: 53)

All disordered patients were diagnosed by our clinicians with FD and no concurrent OD during regular clinical routine (i.e. only primary functional dysphonia was considered). Healthy subjects were recruited separately but examined analogous to disordered subjects. Only healthy subjects were included that did not show signs of any voice disorder. This study was approved by the ethic committee of the Medical School at Friedrich-Alexander-University Erlangen-Nürnberg (no. 290_13B); written consent was obtained by all subjects.

### Signal extraction and parameter calculation

**Fig 2:**
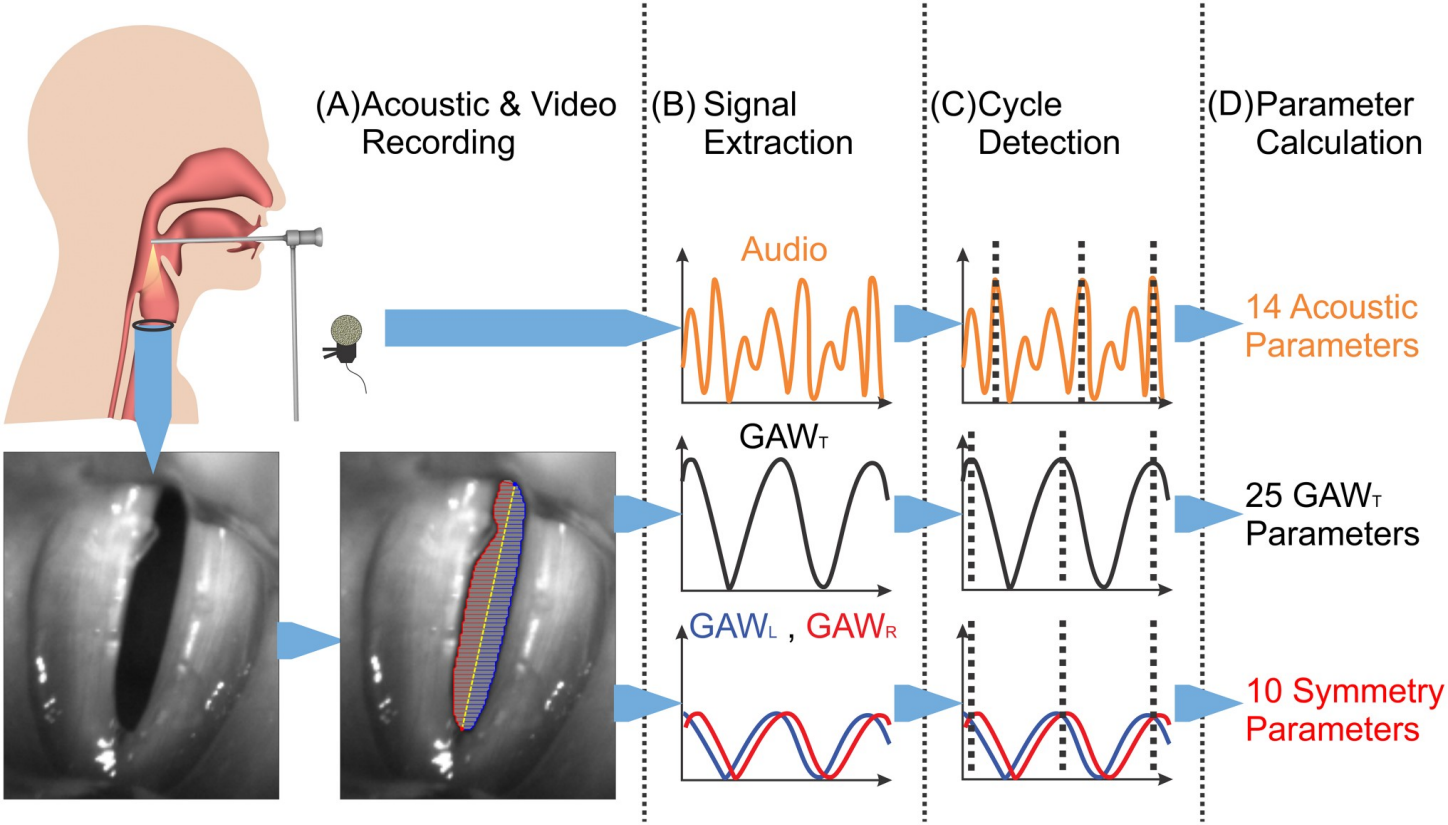
Parameter calculation in four steps. (A) Segmentation of the glottal area between the vocal folds and subdivision in left and right half. (B) Extraction of GAW_R_ (blue), GAW_L_ (red) and GAW_T_ (black) and synchronous audio recording. (C) Detection of maximum based cycles in all signals (all GAWs use cycles based on GAW_T_). (D) Calculation of 14 acoustic parameters, 25 GAW parameters and 10 symmetry, i.e. GAW_L_ and GAW_R_, based parameters.

High-speed video data were processed using a preliminary version of the in house developed software Glottis Analysis Tools (GAT-2020), being freely available upon request. It is the next version of GAT-2018, and includes several bug fixes and an improved cycle detection algorithm. The process of segmentation and parameter calculation is illustrated in Fig 2. For a detailed explanation of the segmentation process see [38]. GAWs describing the total glottal area (GAW_T_) and the left and right half of this glottal area (GAW_L_ and GAW_R_) were extracted from HSV videos. The acoustic signal was synchronously recorded using a clip microphone. Maximum based cycles (i.e. each cycle starts at a sufficiently distinct local maximum and ends before the next one) were detected in GAWs and acoustic signals. From all parameters featured in the GAT-software a set of relevant parameters, based on previous work [28, 29, 38, 39], was selected. Only parameters were included that were previously found to be resistant towards certain influencing factors (spatial resolution and sequence length) [28, 29], mathematically sound [39] and not strongly redundant [38]: 35 GAW- and 14 acoustic-based parameters were considered [40–54].

In Table 2 the parameters used in this study are summarized. "Signal" describes if the parameter was calculated exclusively for GAW or acoustic signal or for both signals. "Averaged" describes if only a single parameter value per signal was calculated or if multiple values were calculated (i.e. mean and standard deviation). Further, abbreviation, parameter unit and source are given. This means that a single row in this table can result in up to four parameters (e.g. for Fundamental Frequency acoustic and GAW based F_0_ [Mean] and F_0_ [Std] were calculated). In S1 Table a more detailed version of Table 2 is given containing names, abbreviations, sources and descriptions of all 49 parameters and, if feasible, formulas.

**Table 2:** Summarized parameter information.

Name	Signal	Averaged	Abbreviation	Unit	Source
**Fundamental period measures**					
**Fundamental Frequency**	both	yes	F_0_	Hz	-
**Perturbation measures**					
**Mean Jitter**	both	no	MJit	ms	[40]
**Jitter (%)**	both	no	Jit(%)	a.u.	[40]
**Period Variability Index**	both	no	PVI	a.u.	[41]
**Mean Shimmer**	both	no	MShim	dB	[40]
**Amplitude Variability Index**	acoustic	no	AVI	dB	[41]
**Energy Perturbation Factor**	GAW	no	EPF	a.u.	[42]
**Symmetry measures**					
**Phase Asymmetry Index**	GAW	yes	PhAI	a.u.	[43]
**Phase Asymmetry**	GAW	yes	PhA	a.u.	[43]
**Spatial Symmetry Index**	GAW	yes	SpSI	a.u.	[43]
**Spatial Symmetry**	GAW	yes	SpS	a.u.	[43]
**Amplitude Symmetry Index**	GAW	yes	AmSI	a.u.	[43]
**Amplitude Symmetry**	GAW	yes	AmS	a.u.	[43]
**Waveform Symmetry Index**	GAW	yes	WaSI	a.u.	[43]
**Glottal dynamic characteristics**					
**Closing Quotient**	GAW	yes	CQ	a.u.	[44]
**Speed Quotient**	GAW	yes	SQ	a.u.	[45]
**Glottis Gap Index**	GAW	yes	GGI	a.u.	[46]
**Plateau Quotient**	GAW	yes	PQ	a.u.	[47]
**Glottal Area Index**	GAW	yes	GAI	a.u.	[48]
**Noise measures**					
**Cepstral Peak Prominence**	both	no	CPP	dB	[49]
**Harmonics-to-Noise Ratio**	both	no	HNR	dB	[50]
**Max. Waveform Matching Coeff**.	both	no	WMC_Max_	a.u.	[51]
**Mean Waveform Matching Coeff**.	both	no	WMC_Mean_	a.u.	[51]
**Normalized Noise Energy**	both	no	NNE	dB	[52]
**Signal-to-Noise Ratio (by Klingholz)**	both	yes	SNR_K_	dB	[53]
**Signal-to-Noise Ratio (by Qi)**	GAW	no	SNR_Q_	dB	[54]

Signal: indicates if the parameter was calculated for the GAW, for the acoustic signal or for both. Averaged: if "no": there was only a single value for each signal. If "yes": multiple values per signal were calculated resulting in mean ([Mean]) and standard deviation ([Std]) of this parameter. Further, abbreviation, unit and parameter source are given.

By definition, the GAW-parameters PhA [Mean], PhAI [Mean] and PhAI [Std] were calculated for minimum based cycles [43]. Custom scripts in Python 3.7 were used to analyze the data and to prepare the figures.

### HSV-acoustic correlations

Linear and non-linear relations between HSV and acoustic parameters were considered separately for females and males. For each gender healthy and disordered groups were merged, since parameters are expected to scatter between healthy and disordered voice subjects; i.e. female group (N_F_ & FD_F_ = 184 subjects) and male group (N_M_ & FD_M_ = 66 subjects).

To investigate the linear relations, Pearson correlation coefficients (PCC) and p-values were calculated between all HSV and acoustic parameters. For investigation of general relations, distance correlation coefficients (DCC) and p-values were calculated. Distance correlation is a measure of dependence between random vectors that is only zero when the vectors are independent and 1 when the vectors are identical. Therefore DCC measures linear and non-linear associations between vectors and, contrary to PCC, cannot obtain negative values. For more information see the work by Székely, Rizzo and Bakirov [55]. The p-values calculated for the DCCs are, analogous to PCC p-values, the probability of a correlation being equal or greater than the observed DCC, if the null hypothesis (both parameters are uncorrelated) is true.

**Fig 3:**
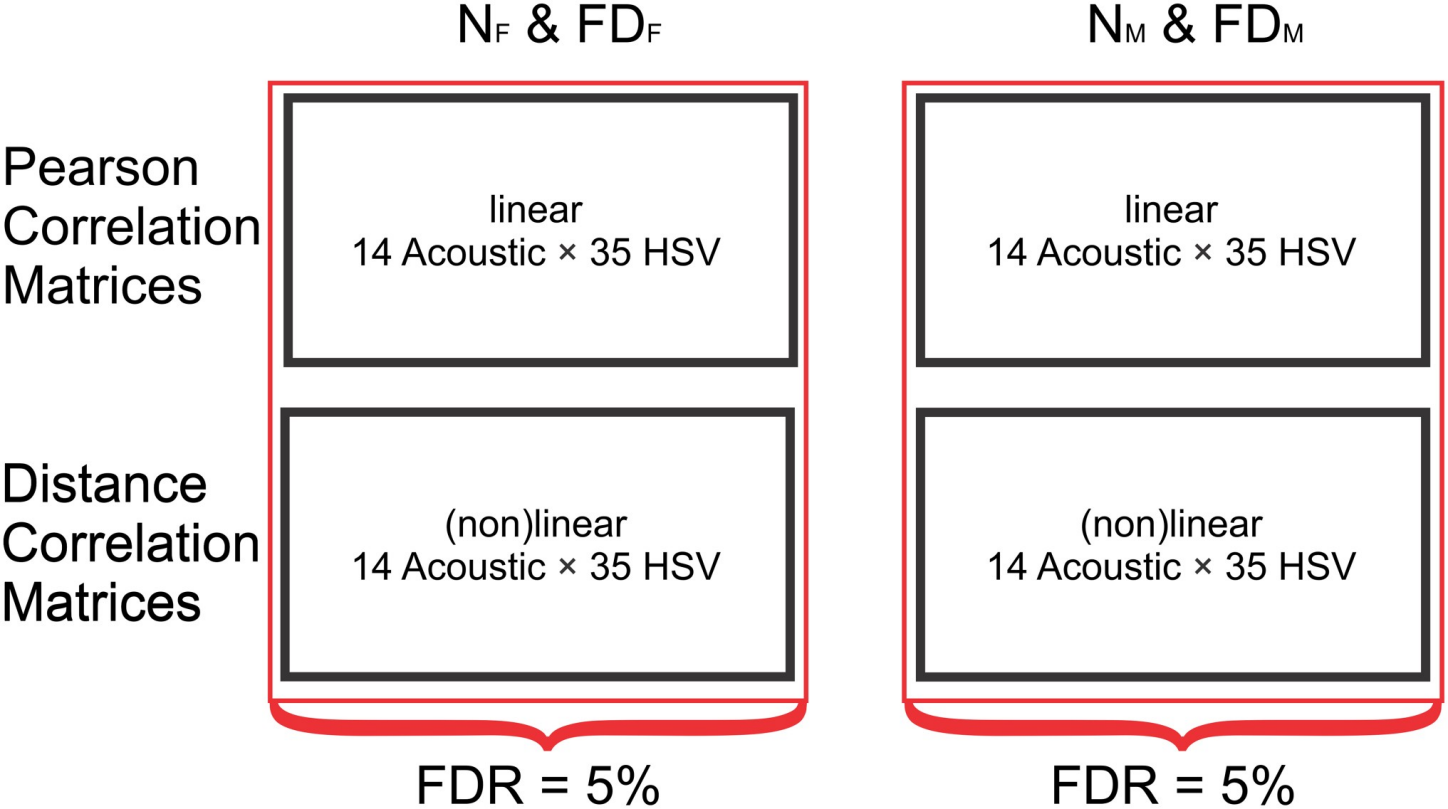
Calculation of Pearson Correlation Coefficients (PCC) and Distance Correlation Coefficients (DCC) between parameters forming Pearson / distance correlation matrices. False discovery rate (FDR) is set to 5% for males and females independently; i.e. FDR is set to 5% for combined subject groups N_F_ & FD_F_ and N_M_ & FD_M_.

This approach yielded two sets of PCCs and two sets of DCCs with respective p-values. We controlled the false discovery rate (FDR), i.e. the expected percentage of false positive tests at 5% using the Benjamini-Yekutieli procedure, since there may be unknown interdependencies between the tests [56]. The p-vales were adjusted accordingly. The entire process is illustrated in Fig 3.

## Results

Three main topics were of interest in this work: (A) Determining the ranges of values for healthy subjects (i.e. females and males with normal voices) and subjects with diagnosis of FD for the investigated parameters, (B) investigating influence of F_0_ on other parameters and (C) detecting relations between parameters not related to the fundamental vocal fold oscillation frequency F_0_.

### Ranges of values for healthy and FD subjects

**Fig 4:**
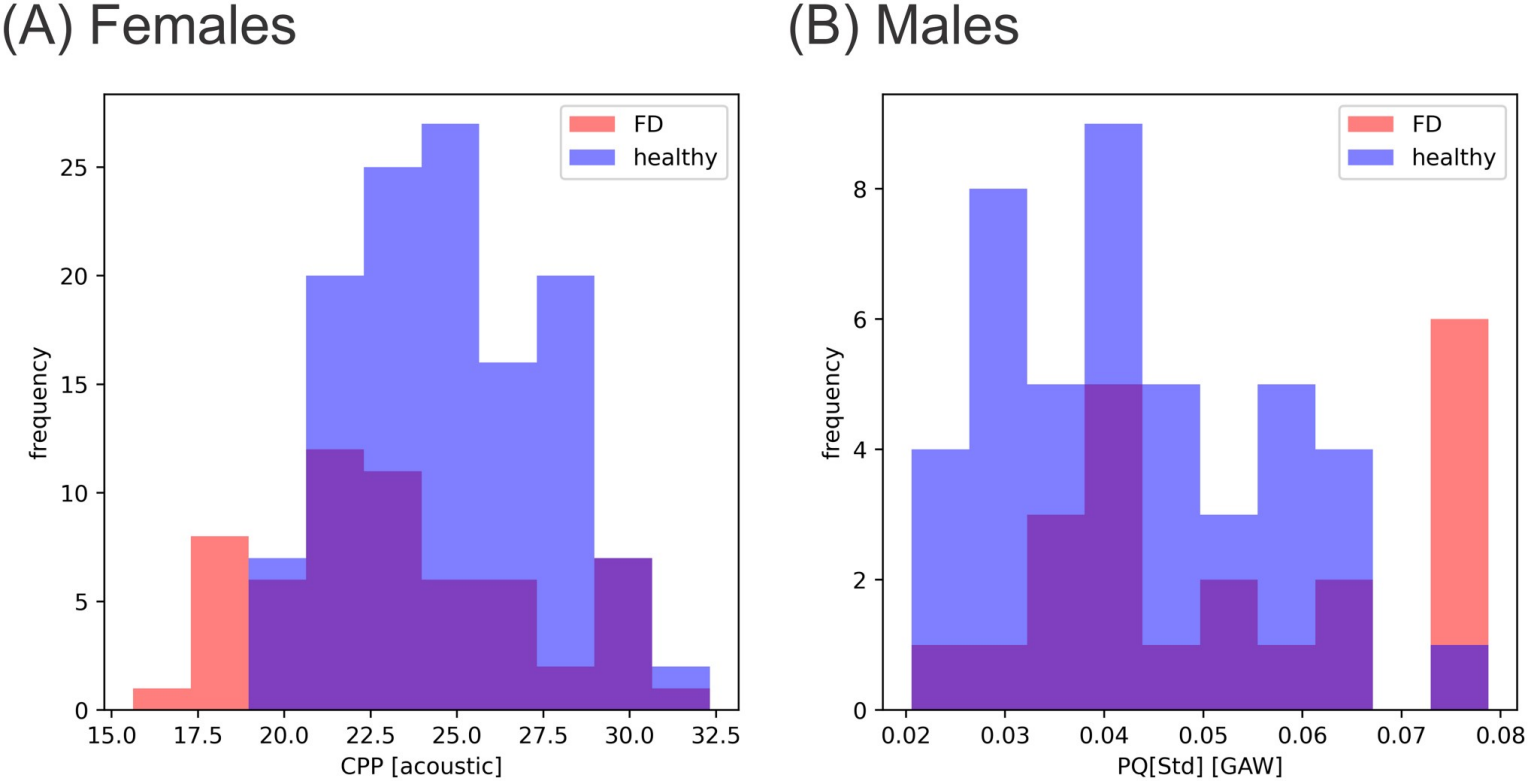
Distribution of parameter values for (A) acoustic based CPP in females and (B) GAW-based PQ[Std] in males. In the violet sections of the histograms blue and red histograms are overlapping.

Statistical values for all four groups (N_F_, FD_F_, N_M_, FD_M_) are collected in S2 Table. This table contains Minimum, Maximum, mean and median-values for these groups as well as the standard deviations, skewness and kurtosis. Further, below this table, distributions of parameter values for all parameters investigated in this study are plotted (similar to Fig 4). Parameter values scattered severely and outliers were common. In Fig 4, exemplary the distributions of two parameters, acoustic based CPP in females and GAW-based PQ [Std] in males, are depicted. Albeit some shift towards lower / higher values may be subjectively identifiable, no strong differences between healthy and FD groups are observable in low order statistical measures like means and medians. However, for some parameters, like GGI [Mean], high order statistical measures (skewness and kurtosis) deviate considerably (see S2 Table). Analogously differences were either similarly small or undetectable in all other GAW- and acoustic-based parameters for both females and males.

### Parameters influenced by *F*_*0*_

We used the rule-of-thumb limits proposed by Mukaka [57] to rate the size of the correlation coefficient (i.e. **absolute value** of PCC or DCC):

- 0.0 ≤ x < 0.3: negligible
- 0.3 ≤ x < 0.5: low
- 0.5 ≤ x < 0.7: moderate
- 0.7 ≤ x < 0.9: high
- 0.9 ≤ x ≤ 1.0: very high

Mukaka only discussed linear relations; however, we also used this limit for distance correlation since it has (in absolutes) the same value range as Pearson correlation. This also leads to better comparability between PCCs and DCCs.

Further, we imposed two conditions that had to be fulfilled to determine a PCC or DCC between two parameters as relevant. (A) The PCC or DCC had to be statistically significant after FDR correction. (B) The PCC or DCC had to be above the rule-of-thumb limit of negligibility for correlation coefficients; i.e. an absolute value greater than or equal to 0.3.

**Fig 5:**
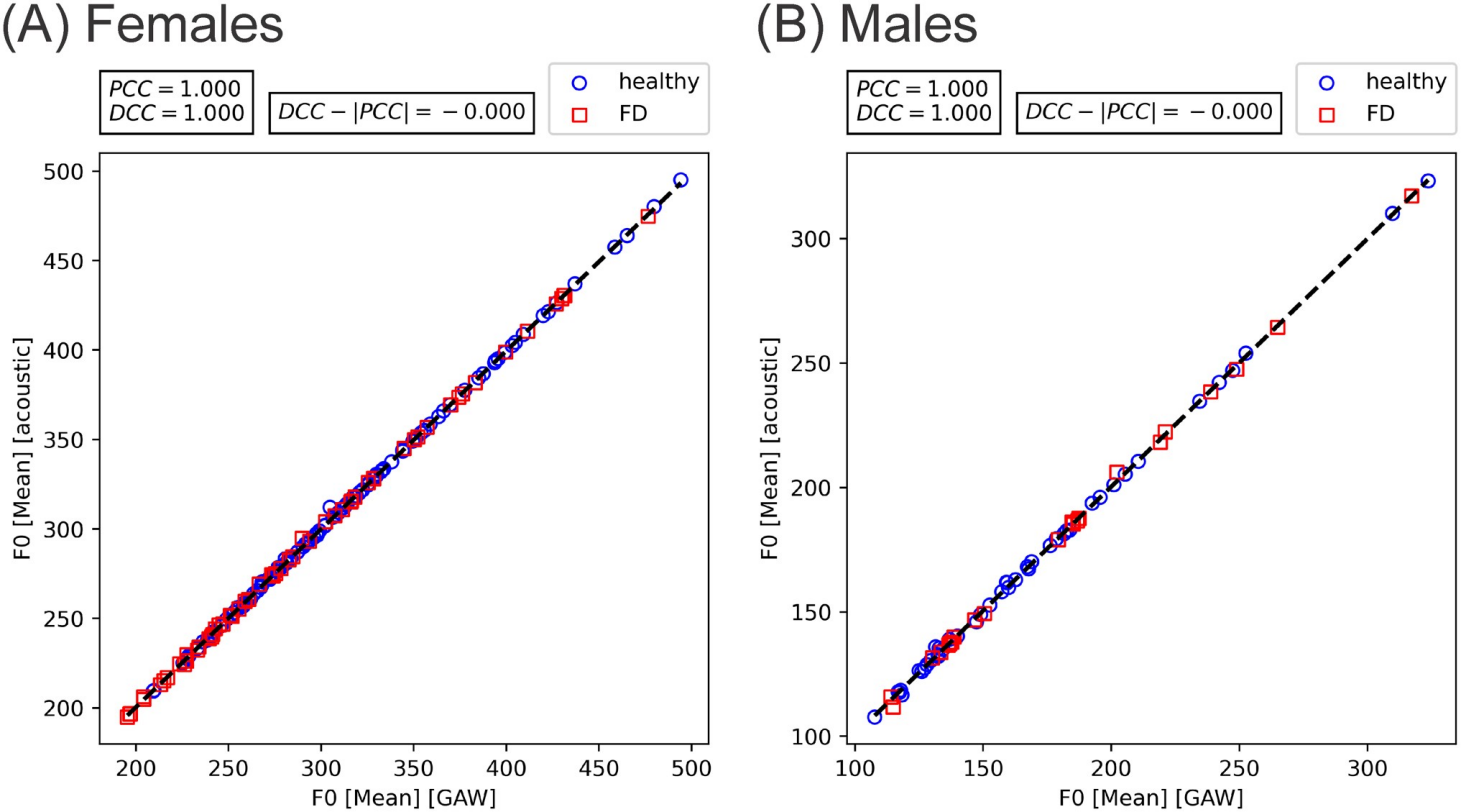
Correlation between GAW and acoustic F_0_ [Mean] in (A) females and (B) males with fitted line (black).

The following relevant correlations were observed: The only parameters that correlated very high (≥ 0.9) were GAW- and acoustic-based F_0_ [Mean], as depicted in Fig 5, for females and males. GAW-based but not acoustic based F_0_ [Std] was highly associated with F_0_ [Mean]. Two parameters were moderately associated with F_0_ [Mean] (PCC or DCC between 0.5 and 0.7). Four parameters showed low and moderate correlations. 15 parameters showed only low correlations (between 0.3 and 0.5). A list of parameters that were associated with F_0_ [Mean], as well as relevant PCCs and DCCs, is provided in Table 3.

**Table 3:** Parameters correlated with GAW-based or acoustic based F_0_ [Mean].

Name	source	PCC females N: 184	DCC females N: 184	PCC males N: 66	DCC males N: 66
**F_0_ [Mean]**	GAW	1.000	1.000	1.000	1.000
**CQ [Mean]**	GAW	0.323	0.352	0.498	0.531
**SQ [Mean]**	GAW	-0.380	0.394		
**PQ [Mean]**	GAW	0.363	0.366		
**HNR**	GAW	-0.393	0.396		
**SNR_Q_**	GAW	0.342	0.414		
**WMC_max_**	GAW	-0.333	0.307	-0.458	
**WMC_mean_**	GAW	-0.385	0.349		
**Mean Shim**	GAW	0.375	0.363		
**EPF**	GAW	0.315	0.303		
**Jit(%)**	GAW	0.400	0.381		
**PVI**	GAW	0.663	0.661		
**F_0_ [Std]**	GAW	0.877	0.860	0.826	0.801
**CQ [Std]**	GAW	0.319	0.413		
**PQ [Std]**	GAW	0.412	0.435		
**PhAI [Std]**	GAW	0.524	0.635	0.598	0.579
**HNR**	acoustic		0.319		
**NNE**	acoustic	-0.420	0.461		
**WMC_max_**	acoustic	0.403	0.502	0.571	0.614
**WMC_mean_**	acoustic		0.383		0.518
**Mean Shim**	acoustic		0.344		
**Mean Jitt**	acoustic	-0.412	0.461	-0.474	0.552
**PVI**	acoustic		0.304		

All statistically significant |PCCs| or DCCs ≥ 0.3 for females and males are given. Values for these correlations can be found in S3 Table. Empty fields are no relevant correlations (below 0.3 or not statistically significant).

**Fig 6:**
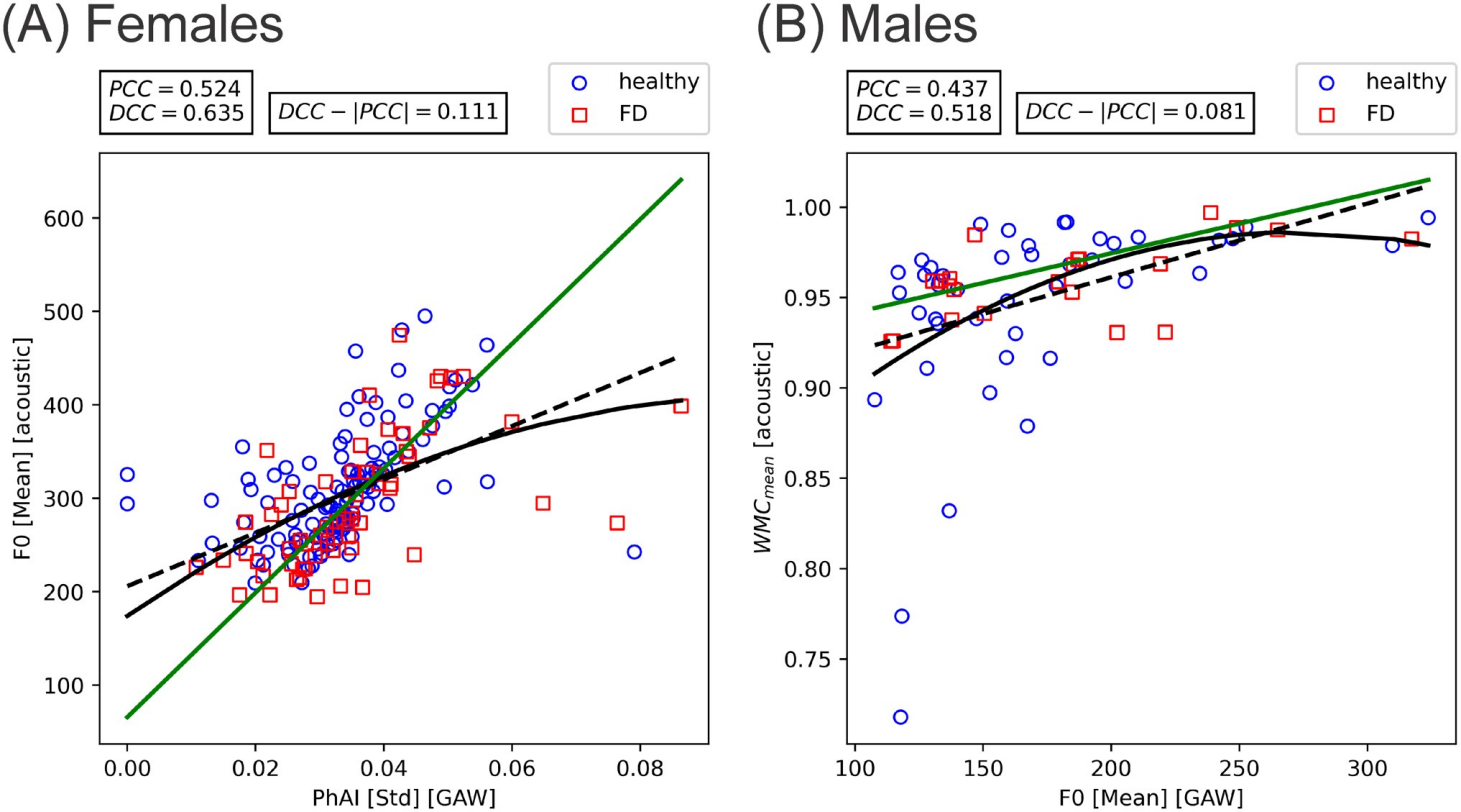
Relevant parameter relations with highest difference between PCC and DCC in (A) females (acoustic based F_0_ [Mean] versus GAW-based PhAI [Std]) and (B) males (acoustic based WMC_Mean_ versus GAW-based F_0_ [Mean]). Fitted are the linear regression line (black, dashed), a second degree polynomial (continuous, black) and a RANSAC regression line (green).

Differences in PCCs (linear correlation) and DCCs (general correlation including linear and non-linear) for the same comparisons were small; i.e. linear correlations are dominant and non-linear relations seem to be small to negligible. For pairings with at least one, PCC or DCC, statistically significant and in absolute values ≥ 0.3, the highest difference in females was 0.111 between GAW-based PhAI [Std] and acoustic-based F_0_ [Mean]. In males the largest difference was 0.081 between GAW-based F_0_ [Mean] and acoustic-based WMC_mean_. In Fig 6, scatter plots for these parameter pairings are depicted, including a fitted regression line and second degree polynomial. Further, a regression line, applying the random sample consensus (RANSAC) algorithm [58], to exclude outlier data points is fitted. As shown in Fig 6, non-linear dependencies between parameters PhAI [Std] / F_0_ [Mean] and WMC_Mean_ / F_0_ [Mean] may exist, but are only weak with large scatter.

No notable differences in PCCs and DCCs between females and males were detected with the exception of GAW-based PVI, related on a moderate level (PCC = 0.663 and DCC = 0.661) with acoustic based F_0_ in females but not in males (no statistically significant PCC or DCC). In general, if for a certain parameter relation a statistically significant PCC or DCC was found in males, the same parameter relation was also statistically significant in females, but not vice versa.

### Correlations excluding mean F_0_

Correlations between GAW- and acoustic-based parameters (excluding F_0_ [Mean]) were in most cases negligible. As shown in Table 4, only 17 low and one barely moderate PCCs or DCCs could be observed. The highest correlations were found between acoustic-based WMC_Max_ and GAW-based F_0_ [Std], which were both also correlated to F_0_ [Mean], Table 3. Analogously to F_0_ [Mean] associated correlations, no distinct differences between PCCs and DCCs in females and males were observable. In S3 Table, all PCCs and DCCs and respective p-values (after FDR-correction) calculated in this study are given.

**Table 4:** GAW-based parameters correlated with the given acoustic based parameter (NNE, CPP, WMC_max_, WMC_mean_ AVI and MJit).

Name	source	PCC females N: 184	DCC females N: 184	PCC males N: 66	DCC males N: 66
**NNE (acoustic based)**					
**F_0_ [Std]**	GAW		0.309		
**CPP (acoustic based)**					
**GGI [Mean]**	GAW	-0.322	0.319		
**GAI [Mean]**	GAW	0.342	0.336		
**WMC_max_ (acoustic based)**					
**F_0_ [Std]**	GAW		0.373	0.489	0.511
**WMC_mean_ (acoustic based)**					
**NNE**	GAW	-0.345	0.339		
**SNR_Q_**	GAW	0.332	0.311		
**AVI (acoustic based)**					
**NNE**	GAW	0.412	0.396		
**CQ [Std]**	GAW	0.320			
**GGI [Std]**			0.310		
**MJit (acoustic based)**					
**PhAI [Mean]**	GAW	0.309			
**F_0_ [Std]**	GAW		0.335		

All statistically significant |PCCs| or DCCs ≥ 0.3 for females and males are given. Empty fields are not relevant correlations (below 0.3 or not statistically significant). Values for these correlations can be found in S3 Table.

## Discussion

For none of the investigated parameters healthy and disordered groups are clearly separable by parameter values, as shown in Fig 4 for two example parameters. However, by inspecting high order statistical measures like skewness and kurtosis that describe the shape of the distribution of parameter values for groups N_F_, N_M_, FD_F_ and FD_M_, several differences between subject groups become apparent (see S2 Table for a comparison of statistical values of all parameters). This is not surprising, since FD is an umbrella-term for a variety of voice disorders [6]. Therefore parameters that describe a certain feature of the phonation process may be expressive for certain subcategories of FD, but may not for others. This and high individual physiological variability [7] may lead to the observed outliers and high variability of parameter values in the data. Since the female and male FD groups consist out of subjects with varying conditions, specific parameters may differ from normal values for only some of the FD subjects. This may then lead to changes in the shape of the parameter distribution in comparison to healthy subjects. In summary, single parameters are not suitable for differentiating healthy from FD subjects and multi-parametric approaches are needed as suggested before [38, 59, 60]. However, if not FD in general but subcategories of FD (e.g. psychogenic dysphonia, conversion dysphonia or tension–fatigue syndrome [6, 61]) are investigated, there could be single parameters or smaller sets of parameters that are able to differentiate these subcategories of FD from healthy voices. Therefore, the collected values for FD subjects, as provided in S2 Table, should be considered preliminary (see shortcomings).

As expected [1, 2], GAW and acoustic F_0_ [Mean] are highly correlated, additionally other parameters are also, to some degree, correlated to F_0_ [Mean], see Table 3. Albeit most of these correlations were only low (0.3 to 0.5), this still implies that these parameters change to a small degree with changing F_0_. Exceptions are GAW-based F_0_ [Std] and PhAI [Std], showing no PCC or DCC below "high" (0.7 to 0.9)" respectively "moderate" level (0.5 to 0.7) in females and males (see Table 3).

Only GAW-based F_0_ [Std] but not acoustic based F_0_ [Std] showed the aforementioned strong correlation with F_0_ [Mean]. F_0_ [Std] is calculated from the inverse cycle lengths ($\frac{1}{cycle\;lenght}$) which vary more for the acoustic signal than in the GAW due to noise and the more complex waveform shape of the acoustic signal which complicates the determination of the exact beginning and ending position of cycles. This effect may mask a potential existing correlation between acoustic based F_0_ [Std] and F_0_ [Mean].

PhAI describes the relative phase shift between GAW_L_ and GAW_R_ in one vocal fold oscillation cycle and PhAI [Std] respectively the standard deviation of this parameter, calculated for all oscillation cycles. Therefore the comparatively high positive correlation of this parameter with F_0_ is expected, since with shorter cycles (higher F_0_), the deviation of PhAI relative to cycle length increases. Regarding such effects, it may be the needed to correct for the influence of F_0_ during further use of the affected parameters.

The found, small differences between PCCs and DCCs indicate weak non-linear relations between the investigated GAW and acoustic features, since this implies that the "general association" between parameters that are measured by DCCs are almost completely explainable by "linear association" that are measured by PCCs. As shown in Fig 6, in the parameter pairings with the highest difference between PCC and DCC, no obvious or only weak non-linear dependencies are observable.

Higher values of PCCs and DCCs and simultaneously a lower number of statistically significant PCCs and DCCs in males than in females may be attributable to the smaller number of available male subjects. PCC and DCC between GAW-based PVI and acoustic F_0_ [Mean] differs the most between females and males. This can be attributed to males phonating at lower fundamental frequencies than females [30] and that the higher the F_0_ [Mean], the stronger the association between GAW-based PVI and F_0_ [Mean].

This relation may be to some degree an artefact attributable to the, in comparison to the speed of vocal fold oscillations, limited sampling rate of the GAW. Even though for 4000 fps recording frame rate and vocal fold oscillation frequencies between 80 and 400 Hz [3], each cycle is represented by 27 to 10 data points; i.e. a single data point shift results in up to 10% change of the cycle length. In female GAWs, less data points are contained in each cycle and hence period perturbation measures such as Jit(%) and PVI are artificially increased. MJit is an exception, since it is not normalized and hence would be expected to be higher in males, however, this effect and the one mentioned before level each other out.

Only 11 pairings of parameters in females and 1 paring of parameters in males that did not include F_0_ [Mean] had statistically significant correlations and none of these correlations exceeded 0.5 (see Table 4). Therefore, the direct relation between investigated features of the GAW and the acoustic signal excluding F_0_ is only low at best. However, there may be still some relations for subcategories of subjects that could not be detected. Further, the influences due to modulation of the airflow / acoustic signal in the vocal tract are not reflected by the GAW. Also, the actually 3-dimensional vocal fold oscillations are not entirely reflected by the one dimensional GAW. This means that 2D and 3D oscillatory characteristics of the vocal folds may be better suited to reflect changes in the acoustic signal than 1D-GAW features do [62, 63]. This also aligns with previous findings, that GAW-based parameters are less important for healthy / FD classification tasks than parameters based on a more complex signal describing the vocal fold oscillation pattern (i.e. Phonovibrogram-based parameters) [38].

To summarize, the main gains from this investigation are as follows:

1. Values of investigated parameters for healthy and FD subjects were not clearly separable. A table containing norm values (Minimum, maximum, Mean, median and standard deviation) for all parameters in all four investigated groups are provided (S2 Table). All parameters were obtained from 250 ms long sustained phonation data (vowel /i/).
2. In many cases parameters are correlated with F_0_, which may require a correction for the influence of F_0_ on these parameters in future studies. We provide a comprehensive list of parameters statistically significantly associated with F_0_ (Table 2).
3. Mostly, linear relations were found between GAW and acoustic parameters. Non-linear relations were only subjectively observable and weak. Further, no strong relations between GAW and acoustic signals, excluding F_0_, were found in females or males. This implies that no clear redundancy exists between both signals but also suggests that the GAW may be a too simplified one dimensional representation of the vocal fold oscillations.

### Shortcomings

In this study more females than males have been investigated which influences the comparisons as explained in the discussion section. This imbalance was not avoidable without excluding many female subjects, since the vast majority of our clinical referrals are females, being similar to other clinics [64]. Also, voice pathologies are more common in females than males [65]. Further, subject age differed between healthy and disordered groups. Albeit we found no strong influence of subject age in a previous study [38], the influence of age on voice parameters is well documented in literature [66–68] and may have influenced the results.

FD is a diagnosis of exclusion and hence a broad term uniting a vast amount of different voice disorders that all have varying symptoms and causes [4, 6]. This means that a table of norm values for FD subjects is only of limited utility, since many parameter values describing only certain features of the voice may also be in the normal range for most of the subcategories of FD. Only for specific subcategories of FD, certain parameter values may deviate. In addition, the analyzed phonatory condition was limited to sustained phonation on vowel /i/. Other paradigms as pitch raise or phonating other vowels will have to be investigated in order to analyze if they are more suitable to differentiate between healthy and FD subjects. However, since we only looked for more general relations between parameters and only limited data was available, the distinction of a large number of FD subcategories was not feasible.

The acoustic signal was recorded in a clinical setting using a clip-microphone and hence was often noisy. We addressed this problem by rating all acoustic signals in regard of signal and background noise and only used data with acceptable external noise levels.

The GAW is only a 1-dimensional representation of the vocal fold oscillation process and hence does not describe the whole information contained in the 2D-HSV recordings [20, 69] or the 3D vocal fold oscillations [62]. For further investigations in vocal fold—acoustic relations, Phonovibrogram-based parameters could be also considered, since the Phonovibrogram is a more complex, 2-dimensional representation of the vocal fold oscillations [63].

More signals, parameters and alternating definitions of parameters exist [25] that were not investigated in this study. Also, exact parameter definitions may differ between software tools [70].

## Conclusion

In this study healthy and FD subjects were not separable by single parameter values. Still, we presented S2 Table containing values for male and female, healthy and FD subjects obtained from 250 ms long sustained phonation data (vowel /i/). This table does not rest upon a sufficiently large and diverse number of subjects to be used as a reference for clinical parameter value ranges. However, it can be expanded and supplemented in future studies to eventually lay the fundamentals for the development of software tools that may allow for objective clinical voice assessment and assisting clinicians.

About half of all 49 investigated parameters were found to be correlated statistically significantly with acoustic or GAW-based F_0_ [Mean]. Albeit most correlations were low (between 0.3 and 0.5) this still implies a measurable influence of F_0_ on the affected parameters. We suggest that, if the parameters affected by F_0_ are used in the future, it may be required to correct for the influence of F_0_, at least for the stronger affected parameters PhAI [Std] and F_0_ [Std].

Only low (and in one case barely moderate) correlations between not F_0_-related GAW- and acoustic-based parameters were found in females and males. Although no strong relations between features of the GAW and acoustic signal besides F_0_ could be found in this work, these findings show the gain of synchronous HSV and acoustic recordings, since not much redundancy is present in both signals. Also, based on these only weak relations between acoustic and GAW-parameters, we conclude that other features besides the glottal area (i.e. specific vocal fold oscillation patterns or the vocal tract) may play a more prominent role in determining acoustic characteristics than the GAW.

## Supporting information

S1 Table Names, abbreviations, sources and descriptions of all 49 parameters. (DOCX)

S2 Table Minimum, maximum, mean, median, standard deviation, skewness and kurtosis values for all four groups (N_F_, FD_F_, N_M_, FD_M_) as well as distributions plots for all parameter values. (XLSX)

S3 Table PCCs and DCCs and respective p-values (after FDR-correction) for all paired parameter correlations calculated in this study. (XLSX)
