# Supplementary material for: Interdependencies between acoustic and high-speed videoendoscopy parameters
Source: PLoS One. 2021 Feb 2;16(2):e0246136. doi: 10.1371/journal.pone.0246136 (PMC7853476; doi:10.1371/journal.pone.0246136)
Supplement: S1 Table — (DOCX) [file pone.0246136.s001.docx]

S1 Table: Parameter information table with abbreviations and sources. Per default parameters are calculated exclusively on the GAW. Parameters calculated for GAW and acoustic signals are highlighted in blue. Parameters calculated exclusively for the acoustic signal are highlighted in purple. The table was split in sub tables for better readability.

| **Parameter (unit) and reference** | **Abbreviation** | | **Parameter description** |
| --- | --- | --- | --- |
| **GAW-based measures** | | | |
| 1. **Fundamental period measures (FPM)** | | | |
| *Mean of Fundamental Frequency* (Hz) | | *F0[Mean]* | reciprocal of cycle duration (Averaged for all cycles) |
| *Standard deviation (Std) of Fundamental Frequency* (Hz) | | *F0[Std]* | reciprocal of cycle duration (Std for all cycles) |

F0_i_ = Fundamental frequency in cycle i

T_i_ = period length of cycle i

$${F0}_{i}=\frac{1}{T_{i}}$$

| 1. **Perturbation measures (PM)** | | |
| --- | --- | --- |
| *Mean Jitter* (ms) [40] | *MJit* | Mean deviation in duration between cycle pairs |
| *Jitter (%)*(a.u.) [40] | *Jit(%)* | Normalized mean deviation in duration between cycle pairs |
| *Period Variability Index* (a.u.) [41] | *PVI* | Normalized mean quadratic deviation in duration between each cycle and an average cycle |
| *Mean Shimmer* (dB) [40] | *MShim* | Mean logarithmic ratio of neighboring dynamic ranges (GAW) or amplitudes (audio) |
| *Amplitude Variability Index* (dB) [41] | *AVI* | Mean quadratic deviation in amplitude between each cycle and an average cycle. (Normalized on quadratic amplitude and logarithmized) |
| *Energy Perturbation Factor* (a.u.) [42] ^1^ | *EPF* | Mean normalized deviation in energy between cycle pairs |

N = number of cycles

T_i_ = period length of cycle i

$\bar{T}$ = average of all cycle lengths

A_i_ = dynamic range or amplitude of cycle i

$\bar{A}$ = average of all amplitudes

E_i_ = Signal energy of Cycle i

$MJit= \frac{\sum_{i=1}^{N-1} \left| T_{i}-T_{i-1} \right|}{N-1}$ $Jit\left( \% \right)= \frac{MJit}{\frac{1}{N}\sum_{i=0}^{N-1} T_{i}}\cdot100$

$PVI=\frac{\frac{1}{N}\sum_{i=0}^{N-1} \left( T_{i}-\bar{T} \right)^{2}}{\bar{T}^{2}}\cdot1000$ $MShim= \frac{20}{N-1}\sum_{i=0}^{N-2} \left| {log}_{10}\left( \frac{A_{i}}{A_{i+1}} \right) \right|$

$AVI= {log}_{10}\left( \frac{\frac{1}{N}\sum_{i=0}^{N-1} \left( A_{i}-\bar{A} \right)^{2}}{\bar{A}^{2}}\cdot1000 \right)$ $EPF= \frac{1}{N-1}\sum_{i=1}^{N-1} \left| \frac{E_{i}-E_{i-1}}{E_{i}} \right|\cdot100$

| 1. **Symmetry measures (SM)** | | |
| --- | --- | --- |
| *Mean of Phase Asymmetry Index* (a.u.) [43] | *PhAI[Mean]* | Average absolute deviation in Phase between cycles of GAW_L_ and GAW_R_ ("Indexes" of symmetry measures are side independent^2^) |
| *Mean of Phase Asymmetry* (a.u.) [43] | *PhA[Mean]* | Average deviation in Phase between cycles of GAW_L_ and GAW_R_ |
| *Mean of Spatial Symmetry Index* (a.u.) [43] | *SpSI[Mean]* | Average absolute difference in area of GAW_L_ and GAW_R_ cycles |
| *Mean of Spatial Symmetry* (a.u.) [43] | *SpS[Mean]* | Average difference in area of GAW_L_ and GAW_R_ cycles |
| *Mean of Amplitude Symmetry Index* (a.u.) [43] ^3^ | *AmSI[Mean]* | Average absolute difference in maximal glottal area of GAW_L_ and GAW_R_ cycles |
| *Mean of Amplitude Symmetry* (a.u.) [43] | *AmS[Mean]* | Average difference in maximal glottal area of GAW_L_ and GAW_R_ cycles |
| *Std of Phase Asymmetry Index* (a.u.) [43] | *PhAI[Std]* | Std of absolute deviation in Phase between cycles of GAWL and GAWR |
| *Std of Spatial Symmetry Index* (a.u.) [43] | *SpSI[Std]* | Std of absolute difference in area of GAWL and GAWR cycles |
| *Std of Amplitude Symmetry Index* (a.u.) [43] ^3^ | *AmSI[Std]* | Std of absolute difference in maximal glottal area of GAW_L_ and GAW_R_ cycles |
| *Std of Waveform Symmetry Index* (a.u.) [43] | *WaSI[Std]* | Std of cyclewise difference in overall shape of GAW_L_ and GAW_R_ |

PhA_i_ = Phase Asymmetry in cycle i (analogous for all other symmetry measures)

N = number of cycles, N_i_ = number of frames in cycle i

GA_i_(j) = Glottal area in frame j of cycle i

${GA}_{i}^{L}(j)$= Glottal area of the left half in frame j of cycle i (analogously R indicates the right half)

T_i_ = period length of cycle i

A_i_ = dynamic range of cycle i

${PhA}_{i}= \frac{{argmin}_{j\in\left\{ 0,\ldots,N_{i}-1 \right\}}\left( {GA}_{i}^{L}(j) \right)-{argmin}_{j\in\left\{ 0,\ldots,N_{i}-1 \right\}}\left( {GA}_{i}^{R}(j) \right)}{N_{i}}$ ${PhAI}_{i}= \left| {PhA}_{i} \right|$

${SpS}_{i} = \frac{\sum_{j=0}^{N_{i}-1} {GA}_{i}^{L}\left( j \right)- \sum_{j=0}^{N_{i}-1} {GA}_{i}^{R}\left( j \right)}{\sum_{j=0}^{N_{i}-1} {GA}_{i}\left( j \right)}$ ${SpSI}_{i}= \left| {SpS}_{i} \right|$

${AmS}_{i}= \frac{{max}_{j\in\left\{ 0,\ldots,N_{i}-1 \right\}}({GA}_{i}^{L}\left( j \right))}{{max}_{j\in\left\{ 0,\ldots,N_{i}-1 \right\}}({GA}_{i}^{R}\left( j \right))}$ ${AmSI}_{i}= \left| {AmS}_{i} \right|$

$${WaSI}_{i}=0.5 \cdot\left( 1+\frac{\left\langle{GA}_{i}^{L}\left( j \right),{GA}_{i}^{R}\left( j \right) \right\rangle}{\left\| {GA}_{i}^{L}\left( j \right) \right\|_{2}\cdot\left\| {GA}_{i}^{R}\left( j \right) \right\|_{2}} \right)$$

| 1. **Glottal dynamic characteristics (GDC)** | | |
| --- | --- | --- |
| *Mean of Closing Quotient* (a.u.) [44] | *CQ [Mean]* | Glottis closing time/ cycle duration (Averaged for all cycles) |
| *Mean of Speed Quotient* (a.u.) [45] | *SQ [Mean]* | Glottis opening time/ glottis closing time (Averaged for all cycles) |
| *Mean of Glottis Gap Index* (a.u.) [46] ^4^ | *GGI [Mean]* | Minimum glottal area/maximum glottal area (Averaged for all cycles) |
| *Mean of Plateau Quotient* (a.u.) [47] | *PQ [Mean]* | Duration during which the glottal area has more than 95% of its maximum/cycle duration (Averaged for all cycles) |
| *Mean of Glottal Area Index* (a.u.) [48] | *GAI [Mean]* | Dynamic range/(maximum of glottal area times Open Quotient) (Averaged for all cycles) |
| *Std of Closing Quotient* (a.u.) [44] | *CQ [Std]* | Glottis closing time/ cycle duration (Std for all cycles) |
| *Std of Speed Quotient* (a.u.) [45] | *SQ [Std]* | Glottis opening time/ glottis closing time (Std for all cycles) |
| *Std of Glottis Gap Index* (a.u.) [46] ^4^ | *GGI [Std]* | Minimum glottal area/maximum glottal area (Std for all cycles) |
| *Std of Plateau Quotient* (a.u.) [47] | *PQ [Std]* | Duration during which the glottal area has more than 95% of its maximum/cycle duration (Std for all cycles) |
| *Std of Glottal Area Index* (a.u.) [48] | *GAI [Std]* | Dynamic range/(maximum of glottal area times Open Quotient) (Std for all cycles) |

CQi = Closing Quotient in cycle i (analogous for all other glottal dynamic characteristics)

T_i_ = period length of cycle i

N = number of cycles, N_i_ = number of frames in cycle i

GA_i_(j) = Glottal area in frame j of cycle i

$\left[ C \to O \right]_{i}$ = Length of opening phase in cycle i (during which the glottis opens)

$\left[ O \to C \right]_{i}$ = Length of closing phase in cycle i (during which the glottis closes)

$T_{i}^{open}$ = Length of open phase in cycle i (during which the glottis is open)

$T_{i}^{plateau}$ = Length of the interval in cycle i where the glottal area is greater than 95% of its ……………maximum

${CQ}_{i}= \frac{\left[ O \to C \right]_{i}}{T_{i}}$ ${SQ}_{i}= \frac{\left[ C \to O \right]_{i}}{\left[ O \to C \right]_{i}}$

${GGI}_{i}=\frac{{min}_{j\in\left\{ 0,\ldots,N_{i}-1 \right\}}({GA}_{i}\left( j \right))}{{max}_{j\in\left\{ 0,\ldots,N_{i}-1 \right\}}({GA}_{i}\left( j \right))}$ ${PQ}_{i}= \frac{T_{i}^{plateau}}{T_{i}^{open}}$

${GAI}_{i}= \frac{A_{i}}{{max}_{j\in\left\{ 0,\ldots,N_{i}-1 \right\}}({GA}_{i}\left( j \right))\cdot\frac{T_{i}^{open}}{T_{i}}}$

| 1. **Noise measures (NM)** | | |
| --- | --- | --- |
| Cepstral Peak Prominence (dB) [49] | *CPP* | Describes the difference between the height of the fundamental quefrency peak and the value of a regression line fitted to the cepstrum at the same position.^5^ |
| Harmonics-to-Noise Ratio (dB) [50] | *HNR* | Describes how similar all cycles of a signal are to an averaged cycle. ^5^ |
| Max. Waveform Matching Coefficient (a.u.) [51] | *WMC_Max_* | Calculates similarity between neighboring sections of the signal of equal length. Gives back maximum similarity |
| Mean Waveform Matching Coefficient (a.u.) [51] | *WMC_Mean_* | Calculates similarity between neighboring sections of the signal of equal length. Gives back average similarity |
| Normalized Noise Energy (dB) [52] | *NNE* | Estimates a pure noise signal using sections of the Fourier transformed input signal between the harmonics. Afterwards the Fourier transformed input signal is set in relation to the Fourier transformed pure noise signal. ^5^ |
| Mean of Signal-to-Noise Ratio_Klingholz_ (dB) [53] | *SNR_K_ [Mean]* | Creates an artificial pure harmonic signal in the Fourier domain based on the harmonics in the input signal. Afterwards the spectral energy of the artificial pure harmonic signal and the spectral energy of the input signal are set in relation. (Average for all windows) ^5^ |
| Signal-to-Noise Ratio_Qi_ (dB) [54] | *SNR_Q_* | Estimates a noise signal based on residual signals derived from the input signal via linear prediction filtering. Afterwards the input signal and the noise signal are set in relation. ^5,6^ |
| Std of Signal-to-Noise Ratio_Klingholz_ (dB) [53] | *SNR_K_ [Std]* | Analogous to *SNR_K_ [Mean]* (Std for all windows) ^5^ |

v_k_ = the kth vector partition of the signal. Each partition has the same length as the average …....length of all cycles in the signal

$${WMC}_{Max}^{k}={max}_{1 \leq k \leq N-1} \left( \frac{\left\langle v_{k},v_{k-1} \right\rangle}{\left\| v_{k} \right\|_{2}\cdot\left\| v_{k-1} \right\|_{2}} \right)$$

$${WMC}_{Mean}^{k}={mean}_{1 \leq k \leq N-1} \left( \frac{\left\langle v_{k},v_{k-1} \right\rangle}{\left\| v_{k} \right\|_{2}\cdot\left\| v_{k-1} \right\|_{2}} \right)$$

^1^ in the source material the formula is given as “Perturbation Factor”. The Energy Perturbation Factor in this work is calculated by inserting cycle energies in this original formula. ^2^ Side dependent versions of symmetry measures give information about the direction of an asymmetric behavior. However, since they are calculated cycle-wise and then averaged over all cycles, left and right-sided asymmetries can cancel each other out. For this reason, side independent versions of the measures exist. They can give information about the actual size of an asymmetric effect, but not about the direction (See also [43]).  ^3^AmSI = (min(max[left glottal area], max[right glottal area]))/ (max(max[left glottal area], max[right glottal area]))) ^4^ In the source material the “Glottis Gap Index” is named “Glottis Closure Index”. ^5^ For the calculation of these parameters rather complex algorithms are needed. For instructions and formulas please refer to the cited sources or to the Glottis analysis Tools user guide. ^6^ SNR_Q_ was not calculated for the acoustic signal since in some cases single 1-Frame artefacts were present in the acoustic signal, that did have a severe influence on SNR_Q_ (but not on other parameters).
